# PATIENT AND FAMILY PARTNER OUTCOMES OF THE FAM-FFC TRIAL

**DOI:** 10.1093/geroni/igad104.0301

**Published:** 2023-12-21

**Authors:** Marie Boltz, Jacqueline Mogle, Ashley Kuzmik

**Affiliations:** Pennsylvania State University, State College, Pennsylvania, United States; Clemson University, Clemson, South Carolina, United States; Pennsylvania State University, State College, Pennsylvania, United States

## Abstract

The Fam-FFC trial tested the hypotheses that patients who participated in Fam-FFC would have more return to baseline function, more physical activity, and less delirium, depressive symptoms, and behavioral symptoms of distress, and that care partners would demonstrate increased preparedness for caregiving (primary outcome) and less anxiety, strain, and burden, as compared to participants in the control arm. The likelihood of returning to baseline function across time for Fam-FFC participants was twice that of the control group by the end of 6 months (OR = 2.39, p =.011). Fam-FFC was also associated with fewer behavioral symptoms of distress at six-months (changeintervention = -1.973, SE = .396, p < .0001, d = .467; changecontrol = -.859, SE = .389, p = .028, d = .203); moderate physical activity, depressive symptoms, and delirium severity did not differ by treatment arm. Preparedness for caregiving increased significantly more from 2 months to 6-months (changeintervention=2.4, SE=.439, d=.563; changecontrol=.893, SE=.450, d=.212, overall p = .02), and marginally increased from discharge to six months. (changeintervention= 2.783, SE= 0.44, d=.682; changecontrol=1.695, SE=0.44, d=0.415, overall p =0.08), in the intervention group, with no group differences in anxiety, strain, and burden. Fam-FFC may prevent some of the post-acute functional decline and behavioral symptoms in persons with dementia while increasing FCP preparedness. Future research should focus on sustainability of patient improvements and addressing the chronic stressors associated with the care partner role.

